# Measuring the effect of enhanced cleaning in a UK hospital: a prospective cross-over study

**DOI:** 10.1186/1741-7015-7-28

**Published:** 2009-06-08

**Authors:** Stephanie J Dancer, Liza F White, Jim Lamb, E Kirsty Girvan, Chris Robertson

**Affiliations:** 1 Department of Microbiology, Hairmyres Hospital, Eaglesham Road, East Kilbride, UK; 2 Department of Microbiology, Southern General Hospital, Govan Road, Glasgow, UK; 3 Scottish MRSA Reference Laboratory, Stobhill Hospital, Glasgow, UK; 4 Department of Statistics & Modelling Science, University of Strathclyde, Glasgow, UK

## Abstract

**Background:**

Increasing hospital-acquired infections have generated much attention over the last decade. There is evidence that hygienic cleaning has a role in the control of hospital-acquired infections. This study aimed to evaluate the potential impact of one additional cleaner by using microbiological standards based on aerobic colony counts and the presence of *Staphylococcus aureus *including meticillin-resistant *S. aureus*.

**Methods:**

We introduced an additional cleaner into two matched wards from Monday to Friday, with each ward receiving enhanced cleaning for six months in a cross-over design. Ten hand-touch sites on both wards were screened weekly using standardised methods and patients were monitored for meticillin-resistant *S. aureus *infection throughout the year-long study. Patient and environmental meticillin-resistant *S. aureus *isolates were characterised using molecular methods in order to investigate temporal and clonal relationships.

**Results:**

Enhanced cleaning was associated with a 32.5% reduction in levels of microbial contamination at hand-touch sites when wards received enhanced cleaning (*P *< 0.0001: 95% CI 20.2%, 42.9%). Near-patient sites (lockers, overbed tables and beds) were more frequently contaminated with meticillin-resistant *S. aureus*/*S. aureus *than sites further from the patient (*P *= 0.065). Genotyping identified indistinguishable strains from both hand-touch sites and patients. There was a 26.6% reduction in new meticillin-resistant *S. aureus *infections on the wards receiving extra cleaning, despite higher meticillin-resistant *S. aureus *patient-days and bed occupancy rates during enhanced cleaning periods (*P *= 0.032: 95% CI 7.7%, 92.3%). Adjusting for meticillin-resistant *S. aureus *patient-days and based upon nine new meticillin-resistant *S. aureus *infections seen during routine cleaning, we expected 13 new infections during enhanced cleaning periods rather than the four that actually occurred. Clusters of new meticillin-resistant *S. aureus *infections were identified 2 to 4 weeks after the cleaner left both wards. Enhanced cleaning saved the hospital £30,000 to £70,000.

**Conclusion:**

Introducing one extra cleaner produced a measurable effect on the clinical environment, with apparent benefit to patients regarding meticillin-resistant *S. aureus *infection. Molecular epidemiological methods supported the possibility that patients acquired meticillin-resistant *S. aureus *from environmental sources. These findings suggest that additional research is warranted to further clarify the environmental, clinical and economic impact of enhanced hygienic cleaning as a component in the control of hospital-acquired infection.

## Background

Increasing hospital-acquired infections have generated much attention over the last decade. The public has linked these infections with their experience of dirty hospitals, but the precise role of cleaning in the control of infection remains unknown [1]. Finding evidence for benefit from routine cleaning is difficult because there are no measurable standards available. This means that healthcare environments are assessed by visual inspection only, which may fulfil aesthetic obligations but does not provide a scientific assessment of the infection risk for patients [2].

Meticillin-resistant *Staphylococcus aureus *(MRSA) is one of the main causes of hospital-acquired infection. Hospitals provide a long-term reservoir for MRSA, since it can survive for months in the environment [1,3]. It can be found on general surfaces such as floors and furniture, and on clinical equipment [2,4-6]. Certain sites, such as curtains, beds, lockers and overbed tables, tend to harbour MRSA more frequently than others [7,8]. These sites are often situated right beside the patient [8,9]. For these reasons, MRSA was chosen as an indicator organism in proposed microbiological standards for surface-level hygiene in hospitals [2].

Patients generally acquire MRSA from hands, and it is possible that healthcare workers transmit MRSA via hands after touching contaminated environmental surfaces [5,10]. This transmission cycle could be broken by more conscientious hand-hygiene but it is notoriously difficult to get everyone to clean their hands at the appropriate time [11,12]. When staff are busy, infection control precautions go awry, including hand washing and cleaning of equipment [13]. It has already been suggested that the limitations of hand hygiene could be mitigated by concentrating resources on cleaning hand-touch sites [8,10].

The main aim of this prospective cross-over study was to introduce one additional cleaner into a surgical ward from Monday to Friday and measure the effect on the clinical environment. After 6 months the cleaner was switched to another matched surgical ward so that each ward acted as a control for the other. We also hoped to investigate the transmission pathways between patients and the environment using molecular epidemiological methods.

## Methods

### Study wards

Two surgical wards with endemic MRSA were matched for bed numbers, bed occupancy rates, floor area, staffing levels, case mix, antibiotic consumption, cleaning schedules and state and maintenance of internal fabric. Each ward contains 21 beds, with two side-rooms at the ward entrance and the remaining beds in groups of three to five in open cubicles on either side of the ward. Ward A is an acute male surgical ward situated on the first floor of a Victorian block, and Ward B, directly above, is for females, although each occasionally hosts patients of the opposite sex. Most patients were admitted for general surgical procedures, with occasional vascular and orthopaedic cases. Routine antibiotic prophylaxis for general surgical cases is amoxicillin, gentamicin and metronidazole, with cephalosporins used for patients allergic to penicillin. The wards share clinical staff and cleaning is performed by the same domestic team to a set specification [14]. Bed occupancy rates are routinely monitored by Greater Glasgow & Clyde NHS Trust. In 2005, each ward experienced 20 new cases of MRSA, most thought to be ward-acquired.

### Ward screening

We screened 10 hand-touch sites on both wards every week for 1 year in order to ascertain overall aerobic colony counts, presence of MRSA and meticillin-susceptible *Staphylococcus aureus *(MSSA) [7,8]. Once-weekly screening took place on different days after the ward had received its routine daily clean. Sites chosen were hand-touch sites at bedside areas (patient lockers, overbed tables and bed frames), clinical equipment (patient hoist, infusion pump and blood pressure (BP) stand), sites at the nurses' work station (computer keyboard, desk and patient notes) and a side-room door handle. Patient curtains were not included because it was not possible to replace these outside of routine procedures. Hand-touch sites were sampled from one ward hoist, BP stand and computer keyboard but from different sites representing the remaining items throughout the ward, excluding those in the isolation rooms. Screening was continued for 7 weeks after completion of the study in order to monitor any residual effects.

### Patient screening

Both wards adhered to identical infection control, screening and antimicrobial policies during the year. Any change in routine screening policy would have confounded the effect of cleaning as a single intervention, since it has already been shown to be an effective control mechanism in its own right [15]. Patients awaiting surgery were routinely screened pre-admission or on the ward if they had been transferred from another unit, hospital or nursing home, or had a history of recent hospital admission, prior MRSA or recent contact with MRSA. All patients with newly diagnosed infections were also screened for MRSA, along with other clinically relevant specimens, in order to detect ward-acquired MRSA infection. Such infections were diagnosed by senior surgical staff using national criteria for surgical site, catheter-associated and respiratory infections [16]. Those patients with no prior history of MRSA, who had been on the ward for >48 hours, had evidence of infection, and from whom the laboratory isolated MRSA were regarded as new cases of MRSA infection. These, along with known MRSA cases, were then screened every week until discharge from the ward.

### Management of patients with MRSA

Patients with MRSA were placed in isolation and managed according to local infection control policies. They were screened at carrier sites, started on a 5-day topical clearance regimen (chlorhexidine washes, mupirocin nasal cream and Corsodyl™ throat spray), and treated with appropriate antibiotics. Patients situated nearby were also screened for MRSA after any new case was found on the ward. If there were no isolation rooms available, patients were cohorted or moved to another ward; this included the other study ward. MSSA acquisition and infection were not audited during the study.

### Enhanced cleaning protocol

Enhanced cleaning began on Ward A in July 2006. The study domestic cleaned from 0730 h until 1530 h each weekday, with holiday and sickness cover provided by a colleague. Both cleaners were recruited from the existing domestic pool and trained to clean hand-touch sites. The study specification included all near-patient hand-touch sites two to three times per day, hand-touch sites at the nurses' station one to two times per day and clinical equipment one to two times per day regardless of nurses' cleaning responsibilities. The study cleaner also cleaned all door handles on the ward two to three times per day as well as items such as plastic wall-mounted racks for leaflets, visitors' chairs and hand-touch sites in the ward office and kitchen.

Day-to-day management responsibilities fell to senior nurses but the cleaners remained professionally accountable to the domestic supervisor. Routine ward cleaning continued as usual, including regular quality control audits on environmental cleaning [14]. Routine cleaning comprised 2 to 3 hours each morning, concentrating on floors and bathroom facilities. There was a 'spot-check' performed later on during the day, in case toilets, in particular, required further cleaning attention. Equipment and cleaning consumables were standard NHS issue, stored separately and maintained according to current protocol. Consumables were chiefly CINCH™ detergent (AGMA, Haltwhistle, UK) and water and Tuffie™ wipes (Health Care Services, Nottinghamshire, UK). Disinfectants are not routinely used on these wards. The extra cleaner only operated on one ward at a time for a period of 6 months before being moved to the other ward. Each ward therefore acted as a control for the other. There was no additional cleaning performed out-of-hours, including weekends.

### MRSA patient-days

Total weekly MRSA patient-days were estimated by calculating all MRSA-positive patient-days each week for each ward [13]. This was done by prospective auditing of all microbiological specimens submitted from both wards and knowledge of patient admission and discharge dates throughout the study. In addition, every patient with MRSA infection and/or persistent colonisation was screened weekly until discharge. Given that we did not screen every new admission to the ward, we probably underestimated the number of MRSA patient-days during the study, but this would have been consistent for both wards and study periods given the cross-over design. All cases of MRSA infection were identified promptly, whether acquired on the ward or before admission to the ward. Patients with MRSA infection are known to shed the organism into the environment more readily than those who are merely colonised [5].

### Microbiology

Clinical samples were processed in the clinical laboratory according to standard operating procedures. Dipslides (Biotrace^®^, Bridgend, UK) were used for environmental screening; these were coated with Baird-Parker (for staphylococci) and nutrient agars (for aerobic colony counts) [7,8,14,17]. Colonial growth was subjected to both quantitative (cfu/cm^2^) and qualitative assessment [2]. One dipslide was used to screen adjacent sites, with each side pressed against the site for 5 seconds at a pressure of approximately 25 g/cm^2 ^[18].

After sampling, dipslides were incubated at 30°C in air for 48 hours. Microbial growth on nutrient agar was quantified as <2.5 cfu/cm^2^, 2.5 to 12 cfu/cm^2^, 12 to 40 cfu/cm^2^, and 40 to 100 cfu/cm^2^, according to the manufacturer's recommendations. Black colonies on Baird Parker were identified to genus level by colonial morphology and Gram film. Gram-positive cocci were differentiated by catalase test and staphylococci designated *S. aureus *or coagulase-negative staphylococci (CNS) by Staph-plus (Pastorex^®^, Stockport, UK). Coagulase-positive isolates were subcultured onto MRSA chromogenic agar (Oxoid, UK) and incubated overnight at 37°C in air. Antimicrobial susceptibility testing was performed using the Vitek^® ^system (bioMerieux, Basingstoke, UK) standardised in accordance with the Clinical and Laboratory Standards Institute guidelines.

### Scottish MRSA reference laboratory methods

Clinical and environmental MRSA were sent to the Scottish MRSA Reference Laboratory, which confirmed identification by latex agglutination and DNase test. All isolates were subjected to PCR detection of *S. aureus *species-specific *nuc *gene, followed by PCR for the *mecA *gene [19,20]. Pulsed-field gel electrophoresis (PFGE) typing of *Sma*I- (Invitrogen, UK) digested DNA was performed by a modification of a previously described method [21]. Briefly, *S. aureus *colonies from overnight cultures were incorporated into agarose plugs. After bacterial lysis, genomic DNA was digested using *Sma*I. PFGE was performed by clamped homogeneous electric field (CHEF) electrophoresis with a CHEF-mapper system (Bio-Rad Laboratories, California, USA). The fragments were separated with a linear ramped pulse time of 6.8 to 63.8 seconds over a period of 23 hours at 14°C. Gels were analysed using DNA analysis software GelCompar II version 5.1 (Applied Maths, Belgium) using the Dice correlation co-efficient. A dendrogram was generated using UPGMA with a tolerance of 1.5%.

### Hygiene status analysis

The standard set for finding a potential pathogen is <1 cfu/cm^2 ^[2]. For this study, we chose both MRSA and MSSA as indicator organisms [22]. A second standard states that total aerobic colony counts (ACCs) from a hand-touch site should not exceed 2.5 to 5 cfu/cm^2 ^[2,23]. Exceeding these levels suggests insufficient cleaning, masks the presence of a pathogen or implies an increased chance of finding a pathogen with similar epidemiological properties, for example, CNS and *S. aureus *[2]. We therefore defined a hygiene failure as a site with ACC greater than 2.5 cfu/cm^2^.

### Epidemiological and cost analyses

Environmental screening data and cases of hospital-acquired MRSA infection were modelled against routine and extra cleaning periods, MRSA patient-days and bed occupancy rates. Any potential transmission between patients and environment were investigated using molecular typing results, dates of isolation and location. Typing also allowed us to ascertain longevity of unique strains in the environment.

Since the project began in July, this allowed each ward to receive 6 months of enhanced cleaning to encompass both winter and summer months. The project was presented to domestic, nursing and medical staff and to the Trust Infection Control Committee for approval and comments before it was launched. It was approved by the R & D department and Ethics committee at the study hospital.

We calculated the overall costs of new MRSA infections potentially saved by the efforts from an extra cleaner. The study cleaner earned £12,320 pa and one new case of MRSA infection was assumed to cost £9,000 [24,25]. Cost of cleaning consumables was included in the calculation.

### Statistical methods

From previous data we expected a background rate of two to four hygiene failures per week [7]. The study was powered to detect a 50% reduction in hygiene failures over a 6-month period, with powers of 97% assuming a background rate of four per week in the normally cleaned ward, 93% with a background rate of three, and 81% with a background rate of two. We expected to find about 20 new MRSA cases on the control ward over the year, assumed to have been acquired on the ward. This only has a power of about 44% to detect a 50% reduction in the numbers of MRSA cases.

The main test of the hypothesis that extra cleaning has an effect on ACC is based upon a linear regression of the log total ACC with terms of cleaning, ward, period and quarter used to investigate the effect of enhanced cleaning on total ACC. A log transformation was required to satisfy the normality assumption in the linear regression and residual plots were used to verify that this assumption was appropriate. This analysis is more powerful than an analysis of hygiene failures, since ACC is a quantitative measurement and hygiene failure is a categorisation of that. The study was powered in terms of hygiene failures, and the powers for ACC analysis will be higher as it is a quantitative measurement rather than a categorical variable. Binomial logistic regression models with factors for ward, cleaning and site were used to estimate the effect of enhanced cleaning on environmental levels of MRSA/MSSA and hygiene failures. Poisson log-linear regression models were used to estimate the effect of enhanced cleaning on new MRSA infections in a ward adjusting for MRSA patient-days and ward, and interaction tests were used to assess the differences in effect over the two wards. Significance levels of 5% were used for the pre-specified hypotheses and 95% confidence intervals for the estimated effects, which, for the overall effect of enhanced relative to routine cleaning, were adjusted for ward differences.

## Results

### Overall levels of microbial growth

**Figure 1:**
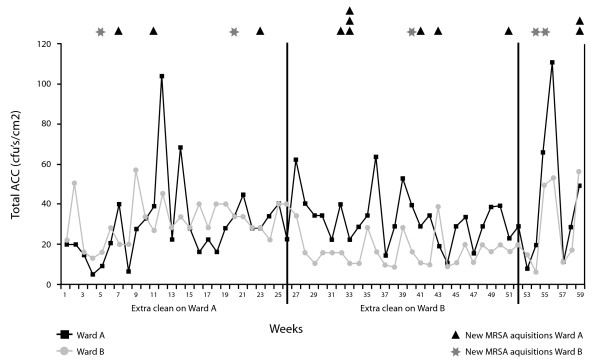
**Total weekly aerobic colony counts from 10 hand-touch sites on Wards A and B demonstrating the effect of enhanced cleaning over two 6-month periods**. New cases of MRSA infections are indicated for each ward; note the cluster occurring on Ward A following withdrawal of the cleaner in the second six months and again on Ward B following completion of the study.

Figure 1 shows the total quantity of microbial growth recovered from both wards over the year and for 2 months after the study finished. New patient MRSA infections are also indicated. ACCs were 32.5% less with enhanced cleaning compared with normal cleaning (*P *< 0.0001; 95% CI 20.2%, 42.9%) (Table 1). Ward B had an overall 19.0% lower ACC than Ward A (*P *= 0.015; 95% CI 4.3%, 31.4%). There was no residual evidence of any temporal effects of period (*P *= 0.58) or quarter (*P *= 0.18). In Ward B, the extra cleaning was associated with a 45.4% reduction in ACC (*P *< 0.0001; 95% CI 33.8%, 55.1%), whereas it was only associated with a 16.5% reduction in ACC for Ward A (*P *= 0.21; 95% CI 36.7% reduction,-10.1% increase).

**Table 1:** Number of environmental MSSA and MRSA isolates, number of new MRSA infections, total and average MRSA patient-days and bed occupancy rates for Wards A and B for two 6-month periods with and without enhanced cleaning.

	**WARD A**		**WARD B**			
	
	**Routine**	**Extra** **Clean**	**Routine**	**Extra** **Clean**	***P*-values**	**Overall difference between extra clean and routine (adjusting for ward)**
No. of environmental MSSA	8	20	22	17	*P *= 0.37	OR^1 ^= 1.25 (0.76, 2.06)

No. of environmental MRSA	9	6	7	6	*P *= 0.45	OR^1 ^= 0.75 (0.35, 1.59)

Mean ACC cfu cm^2^	33.8	31.2	33.4	18.6	*P *< 0.0001	Reduction^2 ^= 32.5% (20.2, 42.9)%

Total no. of sites with ACCs >2.5 cfu cm^2 ^(hygiene fails)	86	73	82	27	*P *< 0.001	OR^1 ^= 0.50 (0.37, 0.66)

Total no. of new patient MRSA infections	7	3	2	1	*P *= 0.032	RR^3 ^= 0.26 (0.08, 0.92)

Average bed occupancy rates	89.7%	90.5%	85.7%	91.8%	*P *= 0.42	Difference^5 ^= 3.5% (-1.7, 8.6)%

Total MRSA patient-days	232	271	95	204	*P *= 0.44	Ratio^4 ^= 1.58 (0.87, 2.88)

Average weekly MRSA patient-days	8.92	10.42	3.65	7.84	As row above	As row above

1 Odds ratio of enhanced cleaning relative to routine cleaning

2 Percentage reduction in total growth when there was enhanced cleaning relative to routine cleaning

3 Relative risk of a new MRSA acquisition when there was enhanced cleaning relative to routine cleaning

4 Ratio of total MRSA patient days when there was enhanced cleaning relative to routine cleaning

5 Difference in bed occupancy percentages when there was enhanced cleaning compared with routine cleaning

MRSA = meticillin-resistant *Staphylococcus aureus*; ACC = aerobic colony count

### Hygiene failures

The rate of hygiene failures with routine cleaning averaged 3.2 per week, as anticipated. Over both periods the enhanced cleaning was associated with a 50.3% reduction in the odds of a hygiene failure (95% CI 33.8%, 62.7% reduction), though there was evidence of a different effect between the two wards, *P *< 0.0001, and the two periods (*P *= 0.0002). In period 1 there were 86 hygiene failures in Ward B (normal cleaning) and 73 in Ward A (extra cleaning), corresponding to a 21.0% reduction (95% CI 45.6% reduction, 14.8% increase) in the odds of having a hygiene failure at a site with enhanced cleaning. In period 2 there were 82 hygiene failures in Ward A (normal cleaning) and 27 in Ward B (extra cleaning), corresponding to a 74.7% reduction (95% CI 84.3%, 59.3%) in the odds of having a hygiene failure with enhanced cleaning. In Ward A, where the extra cleaner was used in the first period, the odds of hygiene failure was 15.3% lower (95% CI 41.8% reduction, 23.5% increase) when the extra cleaner was used. In Ward B, the odds of hygiene failure was 76.5% lower (95% CI 85.4%, 62.1%) when the extra cleaner was used.

### Effect on environmental MSSA and MRSA

MRSA was recovered from 12 sites when the wards received extra cleaning compared with 16 sites when the wards were being routinely cleaned (*P *= 0.45) (Table 1). This was in contrast to the number of MSSA isolates recovered during routine and enhanced cleaning periods, since MSSA was recovered from 37 sites on wards receiving enhanced cleaning and from 30 sites on wards during routine cleaning periods (*P *= 0.37).

### Site contamination of MRSA and MSSA

There was some evidence for different amounts of MRSA/MSSA across the sites (*P *= 0.065) (data not shown). Sites more frequently contaminated with MSSA/MRSA were the bedside locker (17 isolates), overbed table (13 isolates) and bed frame (12 isolates). These are the three sites closest to the patient. There was less contamination on the infusion pump, BP stand and computer keyboard. Whether or not the ward had enhanced cleaning did not affect the site contamination rankings (*P *= 0.52), nor did the ward on which sites were located (*P *= 0.15). The least-contaminated site throughout the study was a touch pad on the infusion pump.

### Bed occupancy rates, MRSA patient-days and number of positive MRSA screens

There was little difference in bed occupancy rates between the two wards throughout the study, although both had higher rates during their respective extra cleaning periods (Table 1). Ward A had bed occupancy rates of 89.7% during routine cleaning and 90.5% during enhanced cleaning; Ward B had rates of 85.7% during routine cleaning months and 91.8% when receiving extra cleaning.

MRSA-positive patient-days ranged from 0 to 25 per week (Table 1). The total number of MRSA patient-days on Ward B during routine cleaning was 95, giving an average of 3.65 MRSA patient-days each week for the 6-month period. During enhanced cleaning, the total number of MRSA patient-days was 204, giving an average of 8 MRSA patient-days per week for this period. Thus, Ward B's clean period experienced more than double the number of MRSA patient-days than the period without extra cleaning. For Ward A, total MRSA patient-days during routine cleaning were 232, giving an average of 9 MRSA patient-days per week. During enhanced cleaning, the total was 271, giving an average of 10.42 MRSA patient-days per week for this period. As for Ward B, total MRSA patient-days were greater for the period without extra cleaning, but not by such a large margin.

The total number of positive MRSA screens on each ward for both periods were examined after removal of duplicates: there were 17 MRSA-positive patients identified on Ward A during the first period (enhanced clean) and 14 during the second period (routine cleaning); on Ward B, there were five MRSA-positive patients during the first period (routine clean) and nine during the second period (enhanced clean). These numbers include the patients who acquired their MRSA on the wards.

### Number of new MRSA infections

During normal cleaning there were a total of nine new cases of MRSA infection on both wards with a total of 327 MRSA patient-days. During enhanced cleaning periods, there were 475 MRSA patient-days overall, leading to an expected 13.1 (= (9/327) × 475) new cases of MRSA occurring during the months the wards were receiving extra cleaning. This assumes that there is a relationship between new MRSA cases and MRSA patient-days, and that enhanced cleaning has the same effect as routine. In fact only four new MRSA infections were observed during enhanced cleaning periods. The rate of new MRSA infections during enhanced cleaning was 26.6% (95% CI 7.7%, 92.3%) of the rate during normal cleaning (*P *= 0.032). It is important to emphasise that the power to detect a reduction in new cases of MRSA was low to start with, and the overall low numbers occurring during the study must be interpreted with caution.

There were no changes in antibiotic policies during the study, nor differences in antibiotic consumption, save for a small increase in use of linezolid on Ward A for complicated MRSA infections. There were no additional hand-hygiene interventions other than routine educational visits from the infection control team including poster reminders. We did not formally audit hand-hygiene compliance.

### Costs

A simple cost analysis was performed. The study cleaner earned £12,320 per annum and consumables were £1,100; this was offset against the number of patients potentially spared MRSA infection. The average cost of one hospital-acquired surgical site infection (SSI) caused by MRSA is estimated as £9,000 [24,25]. If it is correct to assume that five to nine patients were potentially spared MRSA SSI, the hospital saved in the region of £45,000 to £81,000 minus the costs of the cleaner and consumables for one year. This gave overall cost savings of £31,600 to £67,600.

### Molecular typing

**Figure 2:**
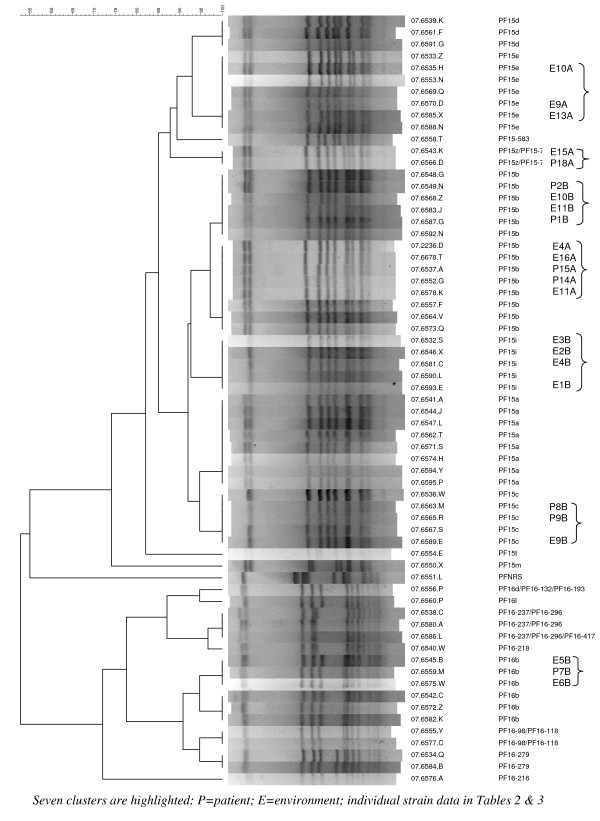
**Dendrogram illustrating genetic diversity among MRSA strains recovered from patients and the environment from Wards A, B and additional ward during the study**. Seven clusters are highlighted: P = patient; E = environment; individual strains are also highlighted in Tables 2 and 3.

Tables 2 and 3 show the PFGE profiles of patient and environmental MRSA strains and their isolation dates. Not all isolates were typed, since some did not survive storage. On Ward A, there were six different PFGE profiles from both patients and the environment (Table 2). Of these, one strain (15b) was recovered from patient notes on 25 October 2006 (E4A), two new patient infections on 14 February 2007 and 18 February 2007 (P14A, P15A), overbed table on 19 February 2007 (E11A) and a BP stand on 21 July 2007 (E16A). Strain 15z/15–71 was isolated from a desk on 4 July 2007 (E15A), followed by a new patient on 12 July 2007 (P18A). The dendrogram suggests a close genetic relationship between these two sets of strains (Figure 2). In addition, there were similar strains isolated from a desk on 13 February 2007, bed on 19 February 2007 and bed again on 22 June 2007 (E9A, E10A and E13A) (Figure 2).

**Table 2:** Date, origin and pulsed-field gel electrophoresis profile of selected patients and all environmental isolates recovered from Ward A.

**Ward A**	**Patient**	**Date**	**Specimen site**	**Environmental** **site**	**PFGE profile**	**Reference** **number**
	P1A	24.7.06	Foot		15e	2217.L
	P2A	10.8.06	Nose		15b/15z/15–71	2218.G
	P3A*	**18.8.06**	**Foot**		**16–237/16–296**	**2220.X**
	P4A	21.8.06	Groin		15 h	2223.N
	P5A	31.8.06	Nose		15a	2226.P
	P6A	08.9.06	Nose		15d	2228.T
	P7A*	**21.9.06**	**Groin**		**15b/15z/15–71**	**2230.P**
		25.9.06		E1A Computer	15–73	2231.F
		06.10.6		E2A Patient notes	15–73	2232.T
		11.10.06		E3A Overbed table	15–73	2233.M
	P8A	13.10.06	Throat		15a	2234.V
	P9A	18.10.06	Heel		15b/15z/15–71	2235.R
		25.10.06		E4A Patient notes	15b	2236.D
		07.11.06		E5A Hoist	16–237/16–296	2237.S
		07.11.06		E6A Door handle	16–237/16–296	2238.Z
	P10A	27.11.06	Throat		15b/15z/15–71	2239.Q
	P11A*	**16.12.06**	**Leg**		**124b**	**2240.D**
	P12A	21.12.06	Central line		16–237/16–296	2241.S
	P13A	05.2.07	Stoma		16–216	6576.A
		13.2.07		E7A Hoist	15a	6541.A
		13.2.07		E8A Bedside locker	15a	6562.T
		13.2.07		E9A Desk	15e	6570.D
	P14A*	**14.2.07**	**Arm**		**15b**	**6552.G**
	P15A*	**18.2.07**	**Catheter site**		**15b**	**6537.A**
		19.2.07		E10A Bed frame	15e	6535.H
		19.2.07		E11A Overbed table	15b	6578.K
	P16A*	**21.2.07**	**Throat**		**16–98/16–118**	**6577.C**
	P17A*	**21.2.07**	**Nose**		**16–218**	**6540.W**
		08.3.07		E12A Bed frame	15a	6595.P
		22.6.07		E13A Bed frame	15e	6585.X
		27.6.07		E14A Hoist	15d	6591.G
		04.7.07		E15A Desk	15b/15z/15–71	6543.K
	P18A*	**12.7.07**	**Throat**		**15b/15z/15–71**	**6566.D**
		21.7.07		E16A BP stand	15b	6678.T
	P19A	23.8.07	Nose		15a	6682.T

PFGE = pulsed-field gel electrophoresis; patient numbers with an asterix denote new acquisitions of MRSA on the ward

**Table 3:** Date, origin and pulsed-field gel electrophoresis profile of selected patients and all environmental isolates recovered from Ward B.

**Ward** **B**	**Patient**	**Date**	**Specimen** **site**	**Environmental** **site**	**PFGE profile**	**Reference number**
		12.7.06		E1B Bedside locker	15i	6593.E
		20.7.06		E2B Overbed table	15i	6546.X
		20.7.06		E3B BP stand	15i	6532.X
		09.8.06		E4B BP stand	15i	6581.C
	P1B*	**11.8.06**	**Wound**		**15b/15z/15–71**	**6587.G**
		25.9.06		E5B Computer	16b	6545.B
		06.10.06		E6B Overbed table	16b	6575.W
	P2B*	**27.11.06**	**Nose**		**15b/15z/15–71**	**6549.N**
	P3B	09.12.06	Sputum		16–237/16–296	2132.V
	P4B	11.12.06	Throat		16–237/16–296	6586.L
		18.12.06		E7B Desk	Pig strain; non-typable	6579.J
	P5B	22.1 07	Throat		15e	6553.N
		19.2.07		E8B BP stand	15e	6588.N
		08.3.07		E9B Infusion pump	15c	6589.E
	P6B	03.4.07	Central line		15e	6533.Z
	P7B*	**11.4.07**	**Nose**		**16b**	**6559.M**
		08.5.07		E10B Door handle	15b/15z/15–71	6568.Z
		22.6.07		E11B Bedside locker	15b/15z/15–71	6583.J
		04.7.07		E12B Hoist	15b/15z/15–71	6557.F
	P8B*	**16.7.07**	**Leg**		**15c**	**6563.M**
	P9B*	**24.7.07**	**Nose**		**15c**	**6565.R**
		21.8.07		E13B Door handle	15a	6680.P

PFGE = pulsed-field gel electrophoresis; patient numbers with an asterix denote new acquisitions of MRSA on the ward

On Ward B, there were four different PFGE profiles from both patients and the environment (Table 3). Of these, two types were isolated from the environment before they appeared as new infections in patients. Type 15c was found from an infusion pump on 8 March 2007 (E9B) before it was isolated from two new patients on 16 July 2007 and 24 July 2007 (P8B, P9B); type 16b was found on a computer keyboard on 25 September 2006 (E5B) and overbed table on 6 October 2006 (E6B) before it was recovered from a patient on 11 April 07 (P7B). Again, the dendrogram suggests a close genetic relationship between these strains (Figure 2). Two further clusters are shown, one involving two new patients (P1B, P2B) on 11 August 2006 and 27 November 2006, a door handle on 8 May 2007 (P10B) and a bedside locker on 22 June 2007 (P11B). The second shows four similar strains from a locker, bedside table and BP stand during the first month of the study (E1-4B).

There were thus indistinguishable strains from patients, which were later found in the environment, and *vice versa *(Figure 2). Indistinguishable strains were recovered from different sites around the ward, sometimes weeks apart [3,5-7]. Not surprisingly, some strains appeared to move between wards. Staff were not screened during this study, however, which means that a carrier role by staff cannot be excluded in any transmission hypotheses [6,7].

## Discussion

This study has demonstrated the effect of one extra cleaner in a prospective cross-over study on two surgical wards. Despite the fact that the cleaning intervention was only delivered on weekdays, it still had a significant impact on the overall levels of microbial contamination from hand-touch sites. The effect was greater on Ward B than for Ward A, one reason for this being the fact that environmental screening was more likely to be performed on a Monday in Ward A during the first few months of the trial. It is possible that the study cleaner did not have time to remove all the accumulated dirt from the weekend. Whilst this creates bias for the results overall, it does provide an indication of the sensitivity of the monitoring method used.

Ward A had the extra cleaner during the first 6 months of the study and the small impact from enhanced cleaning could be evidence of a carry-over effect or an effect of practice. It is likely to be the latter, as there is little evidence of an effect from the extra cleaning in the first 6 months – a reduction of 16.7% (*P *= 0.19; 95% CI 36.2, -8.9%), while there is a large reduction of 45.3% during the second 6 months (*P *< 0.0001; 95% CI 32.8%, 55.5%).

A notable increase is seen in the overall colony counts on Ward A following withdrawal of the cleaner after 6 months (Figure 1). This rebound effect was also seen on Ward B, on completion of the study. It is possible that the withdrawal of enhanced cleaning increased the risk for patient infection, since there were four new MRSA infections on Ward A 4 weeks after the cleaner left (February 2007) and two new infections on Ward B 2 weeks after the study finished (July 2007). Two of the strains from the first cluster on Ward A and both from Ward B in July were genotypically indistinguishable, so these could have been due to patient-to-patient spread without any help from a contaminated environment (Figure 2). The three strains from Ward A in February were different from each other and from MRSA strains from other patients on the ward. These could have occurred as individual episodes of acquisition from hands after touching contaminated environmental sites. The two new cases on Ward B were not included in the overall results of the study, since they occurred after the study had finished. Even if patient-to-patient transmission did occur, the first patient had to acquire MRSA from somewhere before spreading it to another. For each of the two-patient clusters with indistinguishable strains, an apparently similar strain was identified from an environmental source previously (Figure 2).

Only a small reduction in environmental MRSA was documented during the enhanced cleaning periods (Table 1). From previous studies, once-weekly screening of 10 sites throughout the whole ward would not have recovered many MRSA isolates, so this was expected [13]. Similarly, there was little effect on environmental MSSA, with 30 isolates recovered when the wards were routinely cleaned, and 37 recovered during enhanced cleaning. It is known that one in three people carry, and potentially shed, MSSA, whilst the MRSA carriage rate is currently much lower. A larger study with increased frequency of screening might have provided a clearer result on the effect of extra cleaning on environmental MRSA and MSSA.

There were fewer new MRSA infections on the wards receiving extra cleaning over the year-long study. However, not every patient admitted to the wards was screened for MRSA, so making an association between fewer new infections and extra cleaning requires justification. Support for the association comes from the cross-over design, close matching of the wards and the fact that there were no changes in patient screening during the year; if patients outside of the usual high-risk groups for MRSA were admitted without screening, then this occurred consistently throughout the year and for both wards. In addition, given access to previous MRSA rates on the study wards, we were expecting 20 new cases on each ward during the year-long study. There were 10 cases on Ward A and only three on Ward B. We wondered whether the trial itself had some impact overall on the lower numbers of MRSA infections during the study. Staff were well informed about the study before it started.

Another potential confounder is the number of patients with MRSA on the ward. Greater numbers of patients with MRSA would make it more difficult to control the amount of MRSA in the ward, as well as the possibility that patients could pass it directly to each other. We found that there were actually more MRSA patient-days on both wards during the enhanced cleaning periods, especially for Ward B. This finding reinforces the likely impact of the extra cleaner, given the documented reduction in the number of new MRSA infections during extra cleaning periods. In addition, of all the MRSA strains recovered from patients with new infections on the ward at the same time, just two were regarded as indistinguishable during the study (Patients 14A and 15A; Table 3 and Figure 2), and two occurred after the study had finished (Patients 8B and 9B; Table 3 and Figure 2). All the other MRSA strains from cases with new infections were different, although we identified known patients with similar strains who had been resident on the ward previously. These patients had been discharged well before new cases had emerged with similar strains, because these wards were acute surgical wards and did not host long-stay patients.

High bed occupancy rates may also be associated with increased risk of MRSA acquisition, but there was little difference in occupancy rates between the wards, whether they were receiving extra cleaning or not (Table 1) [26-28]. Both wards were actually busier during their enhanced cleaning periods. In addition, higher bed occupancy rates are associated with an increased chance of finding higher ACC, including MRSA/MSSA, from hand-touch sites in the clinical environment [7,8]. This might go some way to explain the small effect from the extra cleaning on the amount of MRSA recovered, and the fact that there was actually more MSSA recovered when Ward B, in particular, was receiving extra cleaning.

We directed the study cleaners to clean hand-touch sites on clinical equipment. Routine cleaning adheres to specified procedures for all surface levels and clinical equipment [29]. It appears that general surfaces receive more attention than others when there are time constraints; that is, staff are more likely to clean floors and toilets, than attend to handles, taps and other hand-touch surfaces [7,13,30]. Nurses are responsible for cleaning bedside areas and clinical equipment but they cannot clean these items properly when they are busy. Furthermore, the effects of a contaminated environment erode any benefits from increasing hand hygiene compliance [31]. This further supports extra cleaning for a ward, particularly one with high bed occupancy, high turnover and nurse shortages [10].

There are several reports in the literature documenting the effect of enhanced cleaning, introduced with educational programmes, in order to improve the quality of environmental cleaning [32-34]. These studies all report success in reducing environmental contamination from hospital pathogens, and one, using the presence of vancomycin-resistant enterococci as hygiene indicator, also demonstrated a reduction in the number of patients colonised with this organism [32]. A more recent study describes the effect of vapourised hydrogen peroxide on *Clostridium difficile *in the environment [35]. The results indicated a significant reduction in environmental contamination of *C. difficile *and in the incidence of *C. difficile *infection on five wards. The findings from these studies support the results presented here, in that the environment plays an important role in the acquisition of hospital infection and that cleaning or other decontamination methods protect patients from hospital pathogens [32-35].

## Conclusion

In summary, this study has shown that one additional cleaner on two surgical wards over 1 year can have an impact on the microbial contamination of high-risk hand-touch sites. Molecular epidemiological methods supported the possibility that patients acquired MRSA from these sites. There is a suggestion that the number of new MRSA infections were reduced relative to the level of MRSA patient-days. Further studies on targeted cleaning of hand-touch sites would be justified in terms of the overall costs of managing MRSA.

## Competing interests

The authors declare that they have no competing interests.

## Authors' contributions

SD and CR designed this study. LW organised the environmental screening. JL set up and ran the microbiology database for patient monitoring and KG performed all the molecular typing of patient and environmental isolates. Statistical analysis was completed by CR. SD analysed data and wrote the paper, with comments received from all authors. All authors read and approved the final manuscript.

## Pre-publication history

The pre-publication history for this paper can be accessed here:


